# Multispectral LiDAR Data for Land Cover Classification of Urban Areas

**DOI:** 10.3390/s17050958

**Published:** 2017-04-26

**Authors:** Salem Morsy, Ahmed Shaker, Ahmed El-Rabbany

**Affiliations:** Department of Civil Engineering, Ryerson University, 350 Victoria Street, Toronto, ON M5B 2K3, Canada; ahmed.shaker@ryerson.ca (A.S.); rabbany@ryerson.ca (A.E.-R.)

**Keywords:** multispectral LiDAR, land cover, ground filtering, NDVI, radiometric correction

## Abstract

Airborne Light Detection And Ranging (LiDAR) systems usually operate at a monochromatic wavelength measuring the range and the strength of the reflected energy (intensity) from objects. Recently, multispectral LiDAR sensors, which acquire data at different wavelengths, have emerged. This allows for recording of a diversity of spectral reflectance from objects. In this context, we aim to investigate the use of multispectral LiDAR data in land cover classification using two different techniques. The first is image-based classification, where intensity and height images are created from LiDAR points and then a maximum likelihood classifier is applied. The second is point-based classification, where ground filtering and Normalized Difference Vegetation Indices (NDVIs) computation are conducted. A dataset of an urban area located in Oshawa, Ontario, Canada, is classified into four classes: buildings, trees, roads and grass. An overall accuracy of up to 89.9% and 92.7% is achieved from image classification and 3D point classification, respectively. A radiometric correction model is also applied to the intensity data in order to remove the attenuation due to the system distortion and terrain height variation. The classification process is then repeated, and the results demonstrate that there are no significant improvements achieved in the overall accuracy.

## 1. Introduction

With the evolution of airborne LiDAR technology, numerous studies have been conducted on the use of airborne LiDAR height and intensity data for land cover classification [1,2,3,4]. Initial studies have combined LiDAR-derived height surfaces in the format of the Digital Surface Model (DSM) or the normalized Digital Surface Model (nDSM) with multispectral aerial/satellite imagery [5,6]. Other investigations have combined multispectral aerial/satellite imagery with LiDAR height and intensity data [7,8,9]. Since most of the previous studies converted either LiDAR intensity or height data into 2D images, typical LiDAR images such as intensity [2,3,4,7,8], multiple returns [2,7], DSM, the Digital Terrain Model (DTM) [2,4] and nDSM [5,6,8] were created. Furthermore, when the LiDAR intensity data were combined with multispectral aerial/satellite imagery, the NDVI was used [5,6,8]. Traditional supervised pixel-based classification techniques such as maximum likelihood [5,9], rule-based classification [2,3,8] and the Gaussian mixture model [7] were applied. Other studies accounted for the spatial coherence of different objects to avoid the noises in the pixel-based classification results by using object-orientated classification techniques [4,6].

Brennan and Webster [2] used a rule-based classification approach for segmenting and classifying five bands, which were created from LiDAR data, into land cover classes. The five bands include DSM, digital terrain model, intensity, multiple returns and normalized height. The image pixels were first segmented into objects by applying threshold values on mean intensity, the standard deviation of intensity, mean DSM, mean normalized height and mean multiple returns. The segmentation process included four levels, where the image objects were separated in the first level into three classes, namely water, low land and elevated objects. The elevated objects were further discriminated into trees and buildings. The low land objects were separated into additional classes, such as low vegetation, roads and intertidal, until the image objects were segmented into ten classes by the fourth level. The overall classification accuracy was 94% and 98% for ten and seven classes, respectively. However, this study relied mainly on the height of LiDAR data. In addition, the threshold values, which were used in the classification rules, were applied to the object’s mean values (e.g., mean intensity). That was a source of error, especially where building edges and ground returns formed one image object in the normalized height band. Furthermore, some dense coniferous trees exhibited single returns as they could not be penetrated by the laser beam. Those trees were misclassified as buildings because the separation of trees from buildings primarily relied on the multiple returns’ band. 

In [3], a sensitive analysis on eight different LiDAR-derived features from height and intensity for three different sites was exhibited. First, image objects segmentation was conducted based on five generated bands from the LiDAR returns, namely bare soil, first returns, last returns, height and intensity. Second, six features based on height, including mean, standard deviation, homogeneity, contrast, entropy and correlation, in addition to mean intensity and compactness were computed. Finally, a decision tree was used to classify the image objects into five land cover classes and achieved more than 90% overall classification accuracy. It should be pointed out that the classification process relied on three features, mean height, height standard deviation and mean intensity, while the use of other features did not improve the land cover classifications. This might be due to that the tested sites were not complex landscapes (e.g., no interference between trees and buildings). Furthermore, the intensity band was assigned a weight of 0.1 in the segmentation process, while other bands were assigned an equal weight of one. This is because the recorded intensity was manually adjusted during the data acquisition. As a result, the intensity data were inconsistent along the different flight lines, meaning that the intensity data were not sufficiently used in this study.

Antonarakis et al. [4] used a supervised object-orientated approach to classify the terrain into nine classes. Eight LiDAR-derived bands, namely the canopy surface model, terrain model, vegetation height model (VHM), intensity model, intensity difference model, skewness model, kurtosis model and percentage canopy model, were first created. A decision tree was then applied in order to classify three urban area datasets. The used approach brought out more than 93% overall accuracy for the three datasets. However, two essential classes were not considered in the presented approach (roads and buildings), even though some buildings were present in one of the three investigated sites. The VHM was calculated by subtracting the digital terrain model (created from the last return) from the canopy surface model (created from the first return). Some last return values had higher elevations than the first pulse return due to noise in the LiDAR receiver, which affected the calculation of VHM. As a result, this approach could not accurately distinguish between the ground and canopy tops. Another source of error is resulted from the triangulated irregular network interpolation of the LiDAR points to create images, whereas high elevations were recorded on the river surface.

Other investigations have explored the use of LiDAR-derived height surfaces, such as the nDSM with multispectral imagery in land cover classification. Huang et al. [5] incorporated LiDAR-derived nDSM with high-resolution RGB aerial image and near-infrared band imagery. A pixel-based classification method, maximum likelihood, was used to obtain four land cover classes, namely buildings, trees, roads and grass, and achieved an overall accuracy of up to 88.3%. The classification accuracy was further improved up to 93.9% using a knowledge-based classification and correction systems. This technique was based on a set of threshold values applied to the height, height difference, smoothness, anisotropic smoothness, intensity, NDVI, transformed vegetation index, area and shape in order to detect the four land classes. Chen et al. [6] incorporated LiDAR-derived nDSM with Quick-Bird images in order to classify the terrain. First, two bands were derived from Quick-Bird imagery, namely the Normalized Difference Water Index and NDVI, and then combined with nDSM. Second, a hierarchical object-oriented classification method was used, which included image segmentation, and then, threshold values to image objects were applied. The hierarchical classification process achieved an overall accuracy of 89.4% for nine land classes. However, this method could not separate road from vacant land, as these objects exhibit similar spectral and elevation characteristics. Both of the aforementioned studies did not incorporate LiDAR intensity data in their research work. The optical images were resampled to coarser resolution to be consistent with the created LiDAR images that resulted in mixed pixels. These pixels presented more than one land cover and caused classification errors.

Other studies used multispectral imagery with LiDAR data (height and intensity) to take advantage of reflectivity variation from spectrum ranges (e.g., visible and Near Infrared (NIR)) of different land objects. Charaniya et al. [7] generated four LiDAR bands from height, intensity, height variation, multiple returns and the luminance band, measured in the visible spectrum, from aerial imagery. A Gaussian mixture model was then used to model the training data of four classes, including roads, grass, buildings and trees. The model parameters and the posterior probabilities were estimated using the expectation-maximization algorithm. Subsequently, those parameters were used to classify the tested dataset and resulted in overall accuracy of 85%. The results demonstrated that the height variation played an important role in classification, where the worst results were obtained by excluding the height band. Furthermore, the overall accuracy was decreased by excluding the aerial imagery. The use of the difference of multiple returns improved the classification of roads and buildings. However, it decreased the classification accuracy of other terrain covers, because of the misclassification of the grass patches. 

Hartfield et al. [8] combined LiDAR data with a 1-m resolution multispectral aerial image. Two LiDAR bands, namely intensity and nDSM, were generated from the LiDAR data, and NDVI was derived from the multispectral aerial image. Classification and regression tree were tested on the number of band combinations. The combination of LiDAR nDSM, multispectral image and NDVI produced the highest overall accuracy of 89.2% for eight land cover classes. The shadow in the aerial image affected significantly the classification results. In addition, misclassification between the bare ground and herbaceous (grass) classes occurred due to the use of the intensity data. This is because the intensity data needed to be calibrated as reported in [8]. Singh et al. [9] combined Landsat Thematic Mapper (TM) imagery with LiDAR-derived bands, which included intensity, the canopy height model and nDSM. The maximum likelihood classifier was applied to classify land cover into six classes. A number of band combinations was tested considering different resolutions of TM imagery such as 1 m, 5 m, 10 m, 15 m and 30 m. Classification of 1-m resolution TM imagery combined with the three LiDAR bands brought out the highest overall accuracy of 85%. The classification results were affected by two main sources of errors; first, the LiDAR data gaps that contributed to misinterpretation when creating 2D LiDAR images; second, the LiDAR intensity data were not normalized to a standard range.

The effects of radiometric correction of LiDAR intensity data on land cover classification accuracies have been recently demonstrated. Radiometric correction aims to convert the recorded intensity data into the spectral reflectance of the land objects. Based on the radar (range) equation, system and environmental parameters were studied such as flying height, range, incidence angle, sensor aperture size and atmospheric attenuation, to correct the LiDAR intensity data [10,11]. By using the radiometrically-corrected LiDAR intensity data, the overall classification accuracies of urban areas were improved by 7.4% [12], 9.4–12.8% [11] and 3.8–16.5% [13].

## 2. Historical Development of Multispectral LiDAR Systems

In the past few years, numerous attempts have been conducted towards multispectral LiDAR systems. Laboratory-based multispectral LiDAR systems have been developed to collect data at different wavelengths [14,15,16]. Analysis of multispectral LiDAR data, collected from Terrestrial Laser Scanning (TLS) platforms, was conducted in order to retrieve the biophysical and/or biochemical vegetation parameters [17,18,19,20,21]. A few attempts have been reported on multispectral airborne LiDAR, which uses various airborne LiDAR systems and combines different flight missions of the same study area [22,23,24].

Laboratory-based multispectral LiDAR systems have been developed to collect data at wavelengths of 531, 550, 660 and 780 nm [14] and 556, 670, 700 and 780 nm [15], in order to measure the 3D structure of forest canopies. Shi et al. [16] developed a calibration method for the backscatter intensity from a laboratory-based multispectral LiDAR systems operating at wavelengths of 556, 670, 700 and 780 nm. This method accounted for the incidence angle and surface roughness. After that, different vegetation indices were defined and explored, in order to improve the classification accuracy.

Other investigations used TLS platforms to collect multispectral LiDAR data. For instance, a dual-wavelength full-waveform TLS platform was developed by [18], operating at two wavelengths (NIR: 1063 nm; mid-infrared (MIR): 1545 nm). The platform was used to record the full-waveform returned from the forest canopies to measure their three-dimensional structure. The Finnish Geodetic Institute developed a Hyperspectral LiDAR (HSL) system transmitting a continuous spectrum of 400–2500 nm [19]. An outdoor experiment was performed using seven wavelength bands ranging from 500–980 nm in order to discriminate between man-made targets and vegetation based on their spectral response [20]. Douglas et al. [21] designed a portable ground-based full-waveform TLS operating at 1064- and 1548-nm wavelengths. The system was used to collect data in the Sierra Nevada National Forest. Subsequently, and based on that the leaves absorb more strongly at 1548 nm compared to stems, the leaves were discriminated from the woody materials.

For multispectral airborne LiDAR attempts, Briese et al. [22] proposed a practical radiometric calibration workflow of multi-wavelength airborne LiDAR data. Their approach was based on full waveform observations (range, amplitude and echo width), flight trajectory and in situ reference targets. The datasets used in this study were acquired by three flight missions based on the same flight plan within three months. Three RIEGL sensors, namely VQ-820-G (532 nm), VQ-580 (1064 nm) and LMS-Q680i (1550 nm), were utilized as one sensor for each mission. Important observations related to this study can be summarized as follows. First, the RIEGL VQ-820-G was mainly designed to survey seabeds, rivers or lakes, where its scan pattern is an arc-like pattern on the ground. As a result, the data collected using this sensor covered a smaller area with a curved boundary, compared to the other two sensors, which produced linear and parallel scan lines. Second, the in situ measurements of reference targets, which were used in the radiometric calibration, were performed using different sensors under specific conditions (i.e., dry condition at zero angle of incidence). Third, the LiDAR data and the in situ measurements of reference targets were collected at different times (in different seasons from August–December). Thus, the surface conditions at the individual flight missions were not identical. Consequently, the calibrated intensity values were affected, such as the calibrated reflectance 1064-nm wavelength showing higher values than other sensors.

Briese et al. [23] calibrated multi-wavelength airborne LiDAR data acquired using the aforementioned three RIEGL sensors. The LiDAR data were acquired by two flight missions (both with an aircraft equipped with two sensors) within a short time period (i.e., four days) to ensure more stable reflectance behavior of the study site at all wavelengths. The calibrated intensity data collected at 532 nm were quite dark, and also, the data acquired at 1064 nm was brighter compared to the other wavelengths. In this study, no classification process is reported. In addition, the different viewing angle of the RIEGL VQ-820-G with respect to the other two nadir-looking sensors produced LiDAR data with different boundaries. Generally, the surface conditions at the individual flight mission were not identical due to temporal surface changes, atmospheric conditions and the influence of moisture content [23].

Wang et al. [24] demonstrated the potential use of dual-wavelength full waveform LiDAR data for land cover classification. The LiDAR data were acquired by two laser sensors, Optech ALTM Pegasus HD400 (Teledyne Optech, Vaughan, ON, Canada) and RIEGL LMS-Q680i operating (RIEGL Laser Measurment Systems, Horn, Austria) at 1064 nm and 1550 nm, respectively. A radiometric correction model was first applied to the LiDAR data acquired from both sensors. The LiDAR points were then converted into spectral images with 1-m resolution and combined for subsequent processing. Three features were then derived from Optech and RIEGL sensors’ data, namely amplitude (intensity), echo width and surface height. Finally, a supervised classification algorithm, the support vector machine, was used to classify the terrain into six classes, including soil, low vegetation, road and gravel, high vegetation, building roofs and water. Different feature combinations were tested, and overall accuracies of 84.3–97.4% were achieved. The conversion of the 3D LiDAR points into 2D spectral images affected the canopy reflectance information in the spectral images by the objects under the canopy, where the canopy could not be separated from the understory vegetation and soil. This study considered the first return only, extracted from each full waveform, for processing. However, land covers such as trees, building roofs or low vegetation may reflect more than one return. Furthermore, when the RIEGL and Optech amplitude information was tested, the building roofs were not completely separated from soil or low vegetation. One possible reason is that the intensity data came from different missions conducted at different times. Thus, the weather and/or surface conditions change over time, and hence, the same object exhibits different intensity values. As a result, the surface height and echo width were considered the major features for land cover discrimination, while the amplitude information was complementary information [24].

In 2014, Teledyne Optech (Vaughan, ON, Canada) developed the world’s first commercial airborne multispectral LiDAR sensor, which is known as the “Optech Titan”. The sensor offers the possibility of obtaining multispectral active data acquisition at day and night. This facilitates new applications and information extraction capabilities for LiDAR. The sensor operates simultaneously at three wavelengths and acquires point clouds in three channels with different looking angles, namely MIR (1550 nm) in C1 at 3.5° forward looking, NIR (1064 nm) in C2 at 0° nadir looking and green (532 nm) in C3 at 7° forward looking. Specifications of the Optech Titan sensor are provided in Table 1 [25].

Combining multispectral LiDAR data collected at three different wavelengths allows for a higher reliability and accuracy compared to the monochromatic wavelength LiDAR data. A few studies have been conducted on the use of multispectral LiDAR data collected by the Optech Titan for land cover classification. Wichmann et al. [26] studied the spectral patterns of different classes and showed that the intensity values could potentially be used in land cover classification. Raster images were created from the LiDAR intensity and height data, and image classification techniques were then applied [27]. 

In a previous work conducted by the authors, the maximum likelihood classifier was applied to single intensity image, combined three-intensity images and combined three-intensity images with DSM [28]. The overall accuracy of classifying the terrain into six classes was 65.5% when using the combined three-intensity images, compared to a 17% improvement when using single-intensity images. In addition, the overall classification accuracy was improved to 72.5% when using the combined three-intensity images with DSM. Moreover, we derived three spectral indices from the intensity values recorded in the three channels. The spectral indices were tested in an urban area and achieved an overall accuracy of up to 96% for separating the low and high vegetation from built-up areas [29].

The combined use of LiDAR height and intensity data improved the results, in comparison with those obtained through either of the multispectral imagery alone or LiDAR height data (DSM) with high-resolution multispectral aerial/satellite imagery [30]. Although LiDAR systems acquire 3D dense and accurate point clouds, most of the previous studies converted the 3D point clouds into 2D intensity and/or height images, so that image classification techniques can be applied. However, with such conversion, the data lose the third dimension (i.e., the z component), which leads to incomplete and potentially incorrect classification results. In this research work, we aim to present the capability of using multispectral airborne LiDAR data for land cover classification. The objectives of this study are: (1) explore the use of existing image classification techniques in classifying multispectral LiDAR data; (2) develop a method for merging multi-wavelengths LiDAR data; (3) develop an automated method for land cover classification from 3D multispectral LiDAR points; and (4) assess the effect of radiometric correction of multispectral LiDAR data on the land cover classification results. This paper is organized as follows: the methodology, including image- and point-based classification techniques, is explained in Section 3; Section 4 presents the study area and dataset; the land cover classification results are illustrated in Section 5; Section 6 discusses and analyzes the results, and finally, the conclusion is summarized in Section 7.

## 3. Methodology

**Figure 1:**
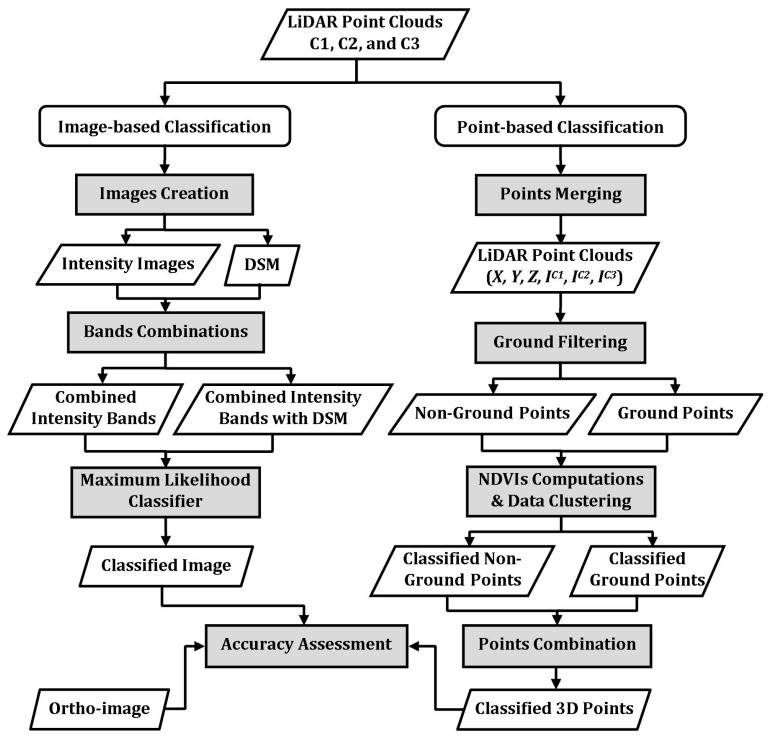
Classification workflow.

Multispectral LiDAR data are used for land cover classification into four classes: buildings, trees, roads and grass. Two independent classification techniques are presented, namely image-based and point-based classification techniques. The workflow of the classification process is shown in Figure 1. The image-based classification technique is based on creating a number of bands from the height and intensity of the LiDAR data. Three intensity images are created from the intensity data collected at the three wavelengths. In addition, a DSM is created from the height data. The three-intensity images are combined together with the DSM. The maximum likelihood classifier is then applied to the combined three-intensity images and the combined three-intensity images with DSM. The point-based classification technique is applied directly to the 3D point clouds. LiDAR points from the three channels are first combined, and the three intensity values are assigned for each single LiDAR point. Subsequently, the ground filtering technique is used to separate non-ground from ground points. Three spectral indices are then computed based on the three intensity values to classify non-ground points into buildings and trees and ground points into roads and grass. These two techniques were applied to raw LiDAR intensity data and radiometrically-corrected LiDAR intensity data. The classification results are validated using an aerial image captured simultaneously with the acquisition of the LiDAR point clouds by the same system. Three accuracy measures are used in the validation, namely overall, producer’s and user’s accuracies, as well as the Kappa statistic.

### 3.1. Image-Based Classification Technique

The workflow of the proposed image-based classification technique starts by creating raster images from LiDAR point cloud data. Three intensity images are created from the recorded intensity values at the three wavelengths. In addition, the points’ elevations are used to create a height image (i.e., DSM). The pixel (cell) size equal to double average point spacing (i.e., 1 m) is selected to ensure a sufficient number of points within the cell. The mean intensity values or elevations were calculated from all points within a pixel and assigned to that pixel. A moving average window 3 by 3 is then used to fill the voids between pixels in the created images [31]. Two band combinations are stacked, namely intensity images from the three wavelengths (combined intensity bands) and intensity images with DSM (combined intensity bands with DSM). After that, training areas for four classes are selected based on an aerial image of the study area to produce a spectral signature for each class. Some classes are composed of separately-sampled classes to account for the variety in the spectral attribute. For instance, buildings class is comprised of samples from various roof colors, such as white roofs, grey roofs and red roofs. Furthermore, samples from high vegetation with different greenness values are included in the trees class. Finally, a supervised classification, the maximum likelihood classifier, is applied to the two band combinations. The maximum likelihood classifier accounts for the probability that a pixel/point belongs to a particular class and considers the variability of classes.

### 3.2. Point-Based Classification Technique

The point-based classification technique is divided into four phases and applied directly to the 3D point clouds. First, the collected point clouds in different channels are combined, and three intensity values for each single point are estimated. Second, non-ground points are separated from ground points based on the elevation attribute using ground filtering technique. Third, NDVI values are computed for non-ground and ground points from intensity values recorded at different channels. Fourth, the Jenks natural breaks optimization method is used to define threshold values and subsequently to cluster the LiDAR points into different classes. More details of this technique are explained in the following sub-sections.

#### 3.2.1. Multi-Wavelength LiDAR Points Merging

**Figure 2:**
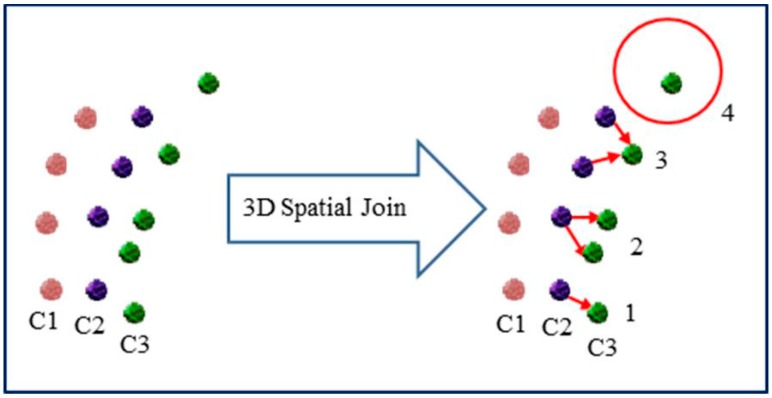
3D spatial join between points from C2 and C3.

As the new multispectral sensor acquires LiDAR data at different wavelengths, point clouds are collected for the same coverage area, but with different intensity values relevant to different wavelength. However, merging those point clouds and predicting the intensity values for each single point at all wavelengths make the available data more dense and reliable. Although Optech Titan operates simultaneously at the three wavelengths, it acquires LiDAR points in the three channels at different angles. Consequently, collected points from the same object in different channels may not coincide completely at the same location. A 3D spatial join technique could provide a possible solution for merging points from all channels, where an intensity value of a point from one channel is assigned to the nearest point from another channel [26]. However, this technique might lead to incorrect matching between points, as shown in Figure 2 and explained through the following scenarios. Case (1) indicates the perfect point matching from Channels C2 and C3. In Case (2), a point from C2 could be matched twice with two different points from C3, as this point is the nearest neighbor to both points. Case (3) shows two possible neighboring points from C2, which have the same distance to a point from C3. Case (4) indicates that no neighboring points from C2 to a point from C3 within a sphere of predefined radius. Therefore, the intensity values of each point cannot be used the same as the intensity value of the nearest point.

**Figure 3:**
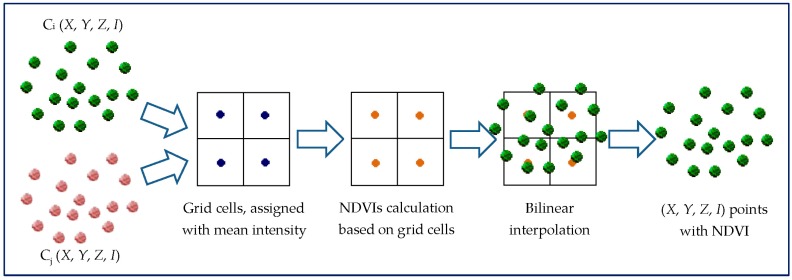
Spectral index calculation from 3D points.

In [29], another method was presented to combine the LiDAR data from the three channels. The LiDAR data from each channel were divided into grids with a cell size of 1 m. The mean intensity value of all points within a cell was assigned to the cell’s center. Three spectral indices’ grids were then calculated using the mean intensity values of the grid cells. The three spectral indices’ values were then interpolated to each LiDAR point using bilinear interpolation from the spectral indices’ grids based on the point’s location, where the adjacent cell centers were used in the calculation. After that, the LiDAR points with the three spectral indices were used for land/water discrimination and land cover classification [29]. This process is summarized in Figure 3.

This method is acceptable when land/water discrimination is required, but it leads to misclassification when classifying the terrain into different land covers. This is primarily due to two reasons. First, the mean intensity values of the points within a grid cell were used. Those points could belong to the same land cover or not, and hence, the cell could represent more than one land cover. Second, bilinear interpolation was used to obtain the spectral values of 3D points. Consequently, the spectral values of points that have multiple returns were incorrectly assigned the same value, such as points from branches and bare soil underneath a tree were assigned the same spectral values. Therefore, in order to correctly predict the intensity value of a point, a median value is calculated from its surrounding points from another channel. The point merging of this research work can be described as follows.

Let *p_i_*, *p_j_* and *p_h_* represent points in C1, C2 and C3, respectively; where *i =* 1, 2, 3, …, *n*_C1_; *j =* 1, 2, 3, …, *n_C_*_2_, *h* = 1, 2, 3, …, *n*_C3_, and *n*_C1_, *n*_C2_ and *n*_C3_ are the total number of LiDAR points collected in C1, C2 and C3, respectively. The LiDAR points in each channel are first organized using a *K-d* tree data structure in order to efficiently apply a multidimensional range search. The neighboring points $N_{p_{i}}^{C2}$ and $N_{p_{i}}^{C3}$ of *p_i_* from C2 and C3, respectively, are obtained within a sphere of predefined search radius (*r*) as follows:

(1) $$N_{p_{i}}^{C2}=\left\{\left(x_{j},\;y_{j},\;z_{j}\right):\sqrt{{\left(x_{j}-x_{i}\right)}^{2}+{\left(y_{j}-y_{i}\right)}^{2}+{\left(z_{j}-z_{i}\right)}^{2}}\leq r\right\}$$

(2) $$N_{p_{i}}^{C3}=\left\{\left(x_{h},\;y_{h},\;z_{h}\right):\sqrt{{\left(x_{h}-x_{i}\right)}^{2}+{\left(y_{h}-y_{i}\right)}^{2}+{\left(z_{h}-z_{i}\right)}^{2}}\leq r\right\}$$

The *r* value is used as 1 m to fulfill two conditions; the first is to have a sufficient number of points, and the second is not to contain any points from different features. The $N_{p_{i}}^{C2}$ and $N_{p_{i}}^{C3}$ points are then arranged in ascending order according to their intensity values. The intensity values $I_{p_{i}}^{C2}$ and $I_{p_{i}}^{C3}$ of *p_i_* from C2 and C3 are calculated, respectively, as:

(3) $$I_{p_{i}}^{C2}=\{\begin{array}{cc} {\left(\frac{N_{p_{i}}^{C2}+1}{2}\right)}^{th}value, & if\;N_{p_{i}}^{C2}\;is\;an\;odd\;number\\ \frac{{\left(\frac{N_{p_{i}}^{C2}}{2}\right)}^{th}value+{\left(\frac{N_{p_{i}}^{C2}}{2}+1\right)}^{th}value}{2}, & if\;N_{p_{i}}^{C2}\;is\;an\;even\;number \end{array}$$

(4) $$I_{p_{i}}^{C3}=\{\begin{array}{cc} {\left(\frac{N_{p_{i}}^{C3}+1}{2}\right)}^{th}value, & if\;N_{p_{i}}^{C3}\;is\;an\;odd\;number\\ \frac{{\left(\frac{N_{p_{i}}^{C3}}{2}\right)}^{th}value+{\left(\frac{N_{p_{i}}^{C3}}{2}+1\right)}^{th}value}{2}, & if\;N_{p_{i}}^{C3}\;is\;an\;even\;number \end{array}$$

The median intensity value is used to avoid any intensity data noise. In case no neighboring points are found, the intensity value is assigned a zero value. Equations (1)–(4) are applied at any point *p_j_* in C2 to obtain the neighboring points $N_{p_{j}}^{C1}$ and $N_{p_{j}}^{C3}$, as well as the intensity values $I_{p_{j}}^{C1}$ and $I_{p_{j}}^{C3}$ from C1 and C3, respectively. The same procedures are applied for any point *p_h_* in C3, where the neighboring points $N_{p_{h}}^{C1}$ and $N_{p_{h}}^{C2}$, as well as the intensity values $I_{p_{h}}^{C1}$ and $I_{p_{h}}^{C2}$ are obtained from C1 and C2, respectively. The LiDAR points are combined, and the duplicated points (*n_d_*) are removed using a MATLAB function “unique”, whereas the unique *xyz* LiDAR points are detected and considered for the classification process; so that the total number of points (*N*) = *n*_C1_ *+ n*_C2_ *+ n*_C3_ − *n_d_*, and each LiDAR point has six attributes: *x, y*, *z, I*^C1^*, I*^C2^ and *I*^C3^.

#### 3.2.2. Ground Filtering

**Figure 4:**
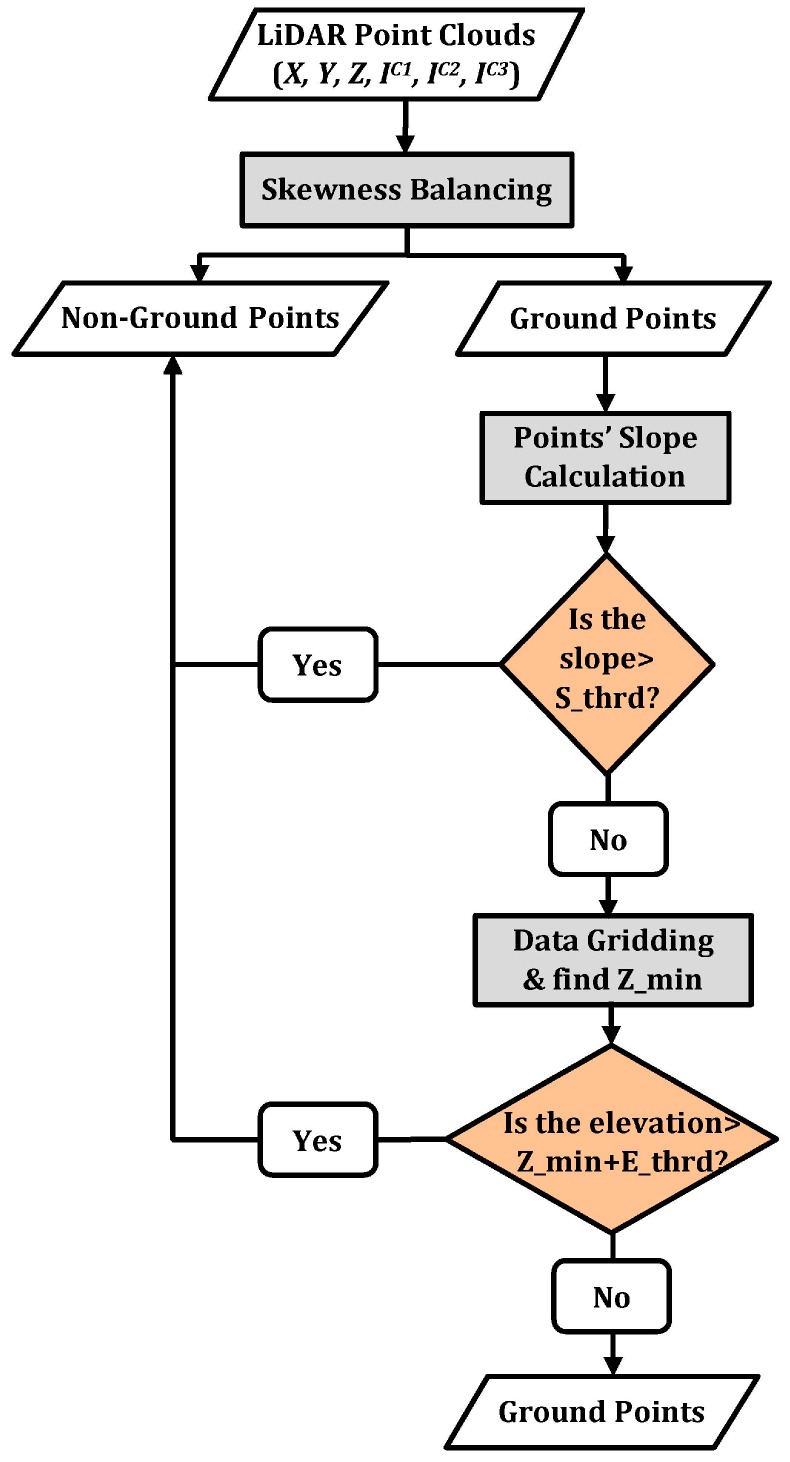
Ground filtering workflow.

The ground filtering aims to separate non-ground points from ground points through the decision rules shown in Figure 4. A statistical analysis algorithm, skewness balancing, was applied to the elevation of points as a first step for ground filtering. The naturally measured data lead to a normal distribution [32]. Thus, the ground points collected within the LiDAR data are assumed to follow the normal distribution, while the other non-ground points (object) may disturb the distribution [33,34]. By removing those non-ground points from the LiDAR data, the ground points are obtained. The higher order moments (e.g., skewness) can characterize the distribution of LiDAR points. The skewness (*Sk*) is defined by:

(5) $$Sk=\;\frac{1}{N\;\cdot \;S^{3}}\;\cdot \overset{N}{\underset{i=1}{\sum }}{\left(Z_{i}-\;\mu \right)}^{3}$$

where *N* is the total number of the LiDAR points, *Z_i_* is the elevation and *i* ∈ {1, 2, …, *N*}, *S* and *μ* are the standard deviation and the arithmetic mean of elevation, defined by Equations (6) and (7) respectively:

(6) $$S=\sqrt{\frac{1}{N-1}\;\cdot \overset{N}{\underset{i=1}{\sum }}{\left(Z_{i}-\;\mu \right)}^{2}}$$

(7) $$\mu =\;\frac{1}{N}\;\cdot \;\overset{N}{\underset{i=1}{\sum }}Z_{i}$$

The elevations of the point clouds are first sorted in ascending order. The skewness is then calculated using Equation (5) from all points. If the skewness is greater than zero, the point with the highest elevation is removed and classified as a non-ground point. The remaining points are used to calculate the skewness, and the process is repeated until the skewness of the point clouds is balanced (*Sk* = 0). After the skewness balancing is performed, the remaining points are classified as potential ground points and assumed to be within a specific slope. As such, the output separation is refined based on the measurement of the slope changes of each LiDAR point with respect to its neighboring points. A threshold value (*S_thrd*) is applied to label the points with a higher slope as non-ground points. Moreover, the remaining ground points are divided into grids to filter out the points with higher elevation. For each grid, a minimum elevation is calculated (*Z_min*), and a threshold value to elevation (*E_thrd*) is applied.

#### 3.2.3. NDVIs Computation 

NDVI values are computed similarly as defined by [35] from the intensity data as follows:

(8) $${\mathrm{NDVI}}_{\mathrm{NIR}-\mathrm{MIR}}=\frac{\mathrm{NIR}-\mathrm{MIR}}{\mathrm{NIR}+\mathrm{MIR}}$$

(9) $${\mathrm{NDVI}}_{\mathrm{NIR}-G}=\frac{\mathrm{NIR}-G}{\mathrm{NIR}+G}$$

(10) $${\mathrm{NDVI}}_{\mathrm{MIR}-G}=\frac{\mathrm{MIR}-G}{\mathrm{MIR}+G}$$

where MIR, NIR and G are the recorded intensity at the MIR, NIR and green wavelengths, respectively. The NDVIs values are between −1 and 1. However, if a point has zero intensity value in two channels, the NDVI will be not a number. In this case, the point is labeled as an unclassified point. 

#### 3.2.4. Data Clustering

The Jenks natural breaks optimization method is used to determine threshold values (*NDVI_thrd*) in order to cluster LiDAR points based on NDVI values [36]. This optimization method has been designed to minimize within-class variances and maximize the between-classes variance. Let the NDVI values range from [*a*, *…*, *b*], where −1 ≤ *a* < *b* ≤ 1 and the threshold value (*NDVI_thrd*) ∈ [*a*, *…*, *b*]. The (*NDVI_thrd*) is identified to cluster the non-ground points into buildings and trees and ground points into roads and grass by maximizing the between-classes sum of squared differences as follows:

(11) $$NDVI\_thrd=arg\;\underset{a\leq t\leq b}{\max}\left\{{\left(M_{1}-M\right)}^{2}\;+{\left(M_{2}-M\right)}^{2}\right\}$$

where *M* is mean of NDVI values, *M*_1_ and *M*_2_ are the mean values of first and second class, respectively. The (*M*) is first calculated. Then, the points are divided into two classes with ranges [*a*, …, *NDVI_thrd*] and [*NDVI_thrd*, …, *b*]. The mean values *M*_1_ and *M*_2_ are calculated. Finally, the optimal threshold value (*NDVI_thrd*) is obtained from Equation (11).

### 3.3. Radiometric Correction

Radiometric correction aims to remove the attenuation due to system- and environmentally-induced distortion. The relationship between the received laser power (*P_r_*) with respect to various system and environmental parameters is described by the radar equation [37]:

(12) $$P_{r}=\frac{P_{t}D_{r}^{2}}{4\pi R^{4}\beta_{t}}\eta_{sys}\eta_{atm}\sigma$$

(13) σ=4πρΑcosθ

where *P_t_* is the transmitted laser pulse energy, *D_r_* is the aperture diameter, *R* is the range, *β_t_* is the laser beam width, *η_sys_* is the system factor and *η_atm_* is the atmospheric attenuation factor. The laser cross-section *σ* consists of the projected target area *A*, the laser scan angle *θ* and the spectral reflectance of the illuminated surface *ρ*. In this research, a radiometric correction model based on the radar range equation is used to remove the system-dependent distortion by converting the (*P_r_*) into the (*ρ*) using *R*, *A* and *θ*, considering that other parameters are constant. The angle *θ* could be used as the incidence angle, which is defined as the angle between the incidence laser beam and the surface normal of any object [11], or a combination between the incidence angle and the scan angle controlled by the surface slope [13]. Further details on the radiometric correction model can be found in [11,13].

## 4. Study Area and Dataset

The study area is located in Oshawa, Ontario, Canada. The Optech Titan multispectral LiDAR sensor was used to acquire LiDAR point for a single strip during a flight mission on 3 September 2014. Optech Titan acquired LiDAR points in three channels at 1075 m altitude, ±20° scan angle, 200 kHz/channel Pulse Repetition Frequency and 40-Hz scan frequency. The mean point density for each channel is 3.6/m^2^, with a point spacing of about 0.5 m. The acquired data consist of trajectory position data, as well as a time-tagged 3D point cloud with multiple returns (up to a maximum of 4 returns) in LASer file format (LAS) for each channel. The LAS data file contains *xyz* coordinates, raw intensity values, the scan angle and the GPS time of each LiDAR point. 

**Figure 5:**
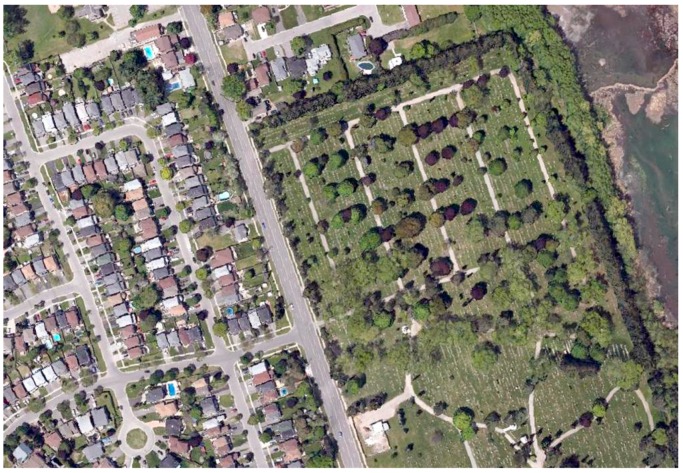
Ortho-rectified aerial image of the study area.

A subset from the LiDAR strip was clipped with a dimension of 550 m by 380 m for testing. The study area covers a variety of land cover features on the ground such as buildings, roads, parking lots, shrubs, trees and open spaces with grass covered. The tested subset has 712,171, 763,507 and 595,387 points from C1, C2 and C3, respectively. The variation in the number of points recorded at different channels depends on the interaction of land objects with different wavelengths (e.g., greenness of the vegetation). An aerial image, captured simultaneously with the acquisition of the LiDAR data, was geo-referenced with the LiDAR data and was used to validate the land cover classification results, as shown in Figure 5. 

Since the 3D reference points are not available, a set of polygons was selected to extract the reference points for each class (i.e., buildings, trees, roads and grass). All points within a polygon drawn on classes, including roads, grass or buildings, were labeled as the same class, while the top layer of the trees class was used as reference points. The total number of points for the four classes was 45,618, distributed as shown in Table 2. 

## 5. Land Cover Classification Results

The study area was classified into four land covers: buildings, trees (includes trees and shrub), roads (asphalt surface, parking lots, and bare soil) and grass (includes green/dry grass and wetland). The accuracy assessment was conducted using a number of reference points within predefined polygons. These polygons were first digitized around the center of the objects to avoid confusion, which might be created by mixed pixels when an image-based classification technique is used. Then, all points within those polygons were labeled from the geo-referenced aerial image. The confusion matrix was then created and the accuracy measures (overall, producer’s and user’s accuracies), as well as the Kappa statistic were calculated.

### 5.1. Image-Based Classification Results

**Figure 6:**
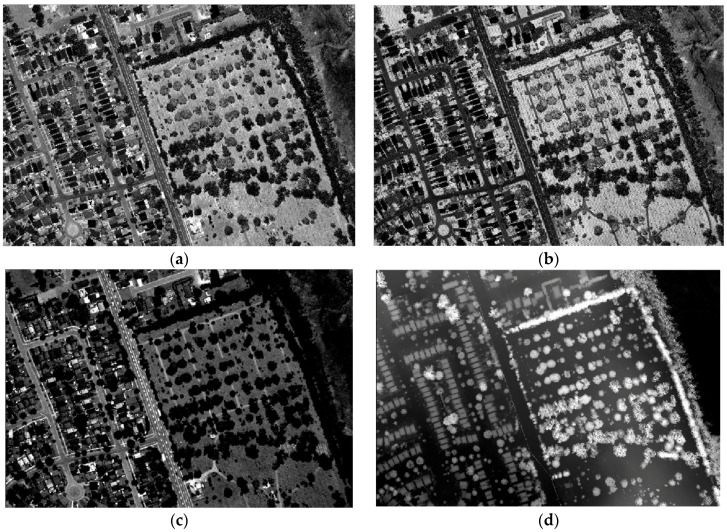
LiDAR raster images: (**a**) C1 intensity; (**b**) C2 intensity; (**c**) C3 intensity; (**d**) DSM.

**Figure 7:**
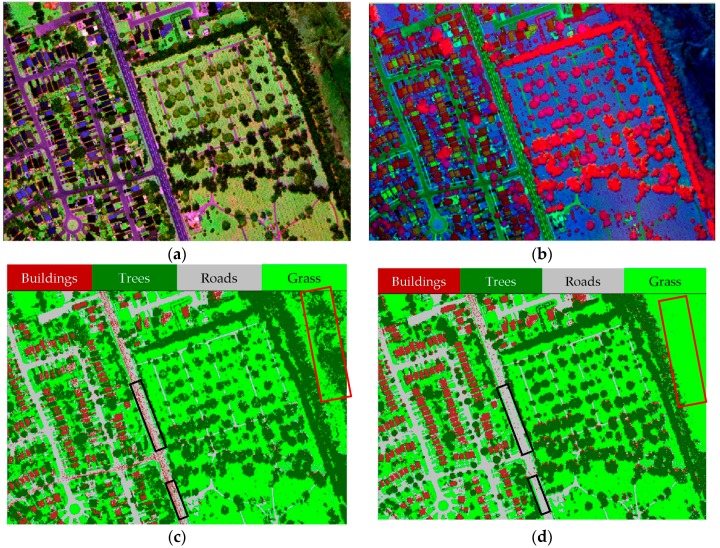
Combined and classified images: (**a**) Combined Intensity Bands (CIBs); (**b**) CIBs_DSM; (**c**) classified image from CIBs; (**d**) classified image from CIBs_DSM.

LiDAR points were used to create three raster images from the intensity values (i.e., C1, C2 and C3 from Channels 1, 2 and 3, respectively) and a raster image from the height data (i.e., DSM), with a spatial resolution of 1 m, as shown in Figure 6. The three raster intensity images were stacked together to compose Combined Intensity Bands (CIBs) and with the DSM raster image (CIBs_DSM). Figure 7a shows a false color composite of the CIBs, which is visualized as C1 in red, C2 in green and C3 in blue; and Figure 7b shows false color composite of the CIBs_DSM, which is visualized as DSM in red, C3 in green and C2 in blue. The maximum likelihood classifier was applied to these two band combinations after identifying training signatures for different classes. Figure 7c,d shows the classified images from combined intensity bands without/with the DSM. The confusion matrix, the overall accuracy and the overall kappa statistics for the two cases are provided in Table 3 and Table 4. The overall accuracy and kappa statistic are 77.3% and 0.675 from the CIBs and 89.9% and 0.855 from the CIBs_DSM.

### 5.2. Point-Based Classification Results

**Figure 8:**
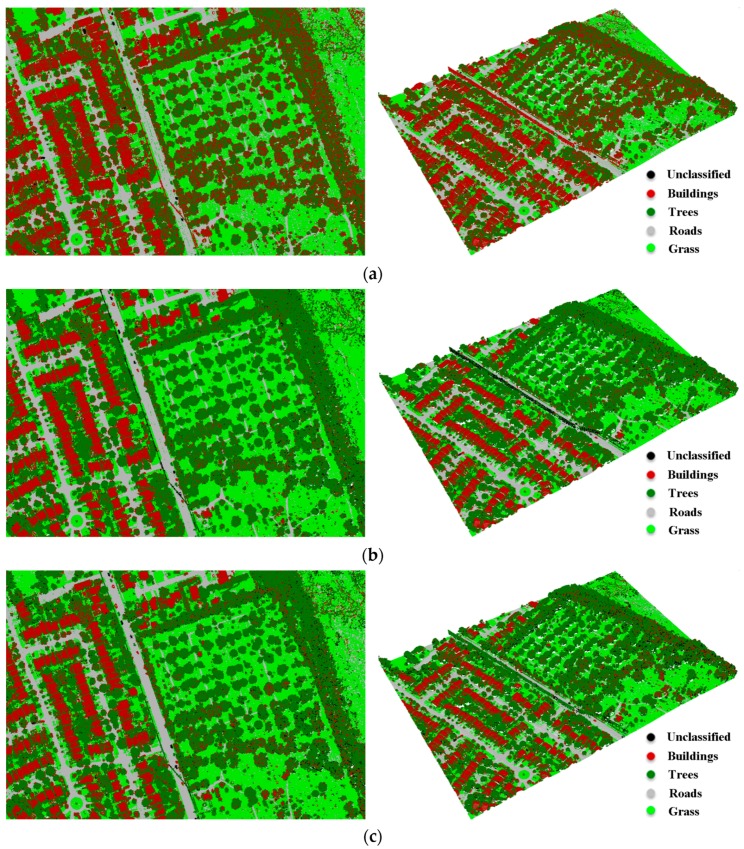
Classified LiDAR points based on: (**a**) NDVI_NIR-MIR_; (**b**) NDVI_NIR-G_; (**c**) NDVI_MIR-G_; (left: (2D view); right: (3D view)).

This technique involved ground filtering and NDVIs computation, which were applied on elevation and intensity attributes, respectively. Subsequently, the points were clustered into different classes. The ground filtering started with skewness balancing in order to separate ground points from non-ground points. The ground points were then refined based on the slope-based change mechanism. The slope of each point with respect to surrounding points was investigated; so that if the slope were greater than a threshold value (*S_thrd =* 10°), the point was classified as a non-ground point. In addition, a few points with higher elevations were not classified as non-ground points. Therefore, the output ground points were divided into grids with a cell size of 25 m. For each grid, if the elevation was greater than 3 m (*E_thrd* = 3 m) above the minimum elevation, the point was classified as a non-ground point. The ground points include roads and grass classes, while the non-ground points include buildings and trees classes. The NDVIs were subsequently computed using Equations (8)–(10). Table 5 shows the threshold values obtained by applying the Jenks break optimization method to the NDVIs for both non-ground and ground points in order to separate buildings from trees and roads from grass, respectively. The vegetation (i.e., trees or grass) has high reflectance at the NIR, MIR and green wavelengths. As a result, the calculated NDVIs of the vegetation points exhibited higher values than the built-up areas (i.e., buildings or roads). Therefore, for a particular point, if NDVI_NIR-MIR_, NDVI_NIR-G_ or NDVI_MIR-G_ ≤ *NDVI_thrd*, the cover type belongs to the buildings or roads class; otherwise, it belongs to the trees or grass class. Figure 8 shows the 3D classified point clouds based on the three NDVIs. The confusion matrix, overall accuracy and kappa statistics for the three cases are provided in Table 6, Table 7 and Table 8. 

There are two sources of errors; the unclassified class and the ground filtering. During the intensity prediction and within the searching radius of any point, if no neighboring points were found, the intensity values were set to zero. As a result, the point’s NDVI was not a number and labeled as an unclassified point. The classification errors due to the ground filtering are highlighted by gray color in Table 6, Table 7 and Table 8, whereas the ground points (roads or grass) were misclassified as non-ground points (buildings or trees) or vice versa.

### 5.3. Radiometric Correction Effect on the Classification Accuracy

The LiDAR intensity data were corrected for the system attenuation (i.e., the range and scan angle). Using Equations (12) and (13), the *ρ* was estimated for each single LiDAR point. The corrected intensity images were then created from the three channels, and the classification process was repeated using the image-based classification technique. The radiometric correction improved the overall accuracy by about 1.7% when using the LiDAR intensity data and by only 0.6% when the DSM was considered. In contrast, the classification accuracy of the buildings class was significantly improved, reaching about 10.8%, and the trees class improved by 3.1%. The same procedures of the point-based classification technique were applied to the radiometrically-corrected LiDAR points; however, no significant improvements in the overall accuracy were recorded. This might be because of the fact that the LiDAR data were collected at a narrow scan angle. 

## 6. Discussion

**Figure 9:**
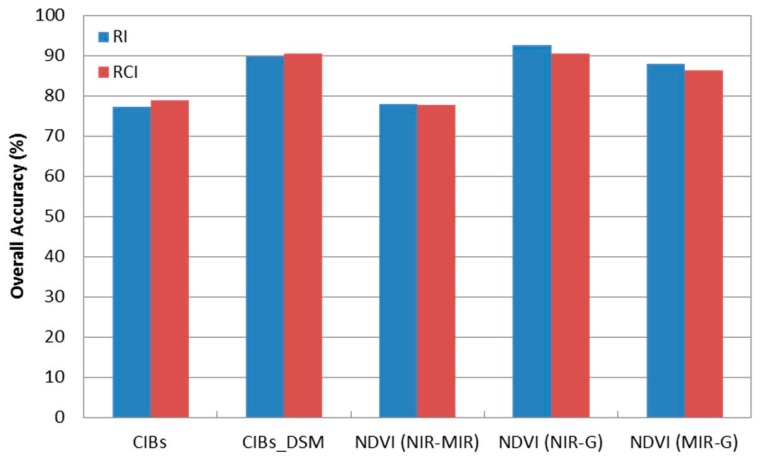
Overall accuracy from the two classification techniques.

Generally, the classification results have demonstrated the capability of using multispectral LiDAR data for classifying the terrain into four classes, namely buildings, trees, roads and grass. The image-based and point-based classification techniques achieved overall classification accuracies of up to 89.9% and 92.7%, respectively. Previous studies achieved overall classification accuracies from 85–89.5% for the same four land cover classes. They used multispectral aerial/satellite imagery combined with nDSM derived from LiDAR data [5,6] or combined with LiDAR height and intensity data [7,8,9], while the presented work in this research used the LiDAR data only. Furthermore, the availability of the multispectral LiDAR data eliminates the need for multispectral aerial/satellite imagery for classification purposes. Figure 9 summarizes the achieved overall accuracy from the two classification techniques.

In particular, the results demonstrated the importance of combining LiDAR intensity and height data, where the overall accuracy increased by more than 12% after the DSM was incorporated with the intensity images. This is clearly notable in Figure 7c, where some pixels (marked by red rectangles) in the study area were misclassified as trees (i.e., high vegetation). Furthermore, many pixels that belong to the road surface were misclassified as buildings, while those pixels were correctly classified as grass (i.e., low vegetation) or roads, after DSM was incorporated as shown in Figure 7d (marked by black rectangles). Significant improvements of the producer’s and user’s accuracies were achieved for the individual land covers when DSM was considered. For instant, an improvement was achieved for the producer’s accuracy of buildings, trees and roads classes by 29.7%, 9.1% and 13.3%, respectively. Furthermore, the user’s accuracy of trees and roads classes was improved by 15.4% and 28.8%, respectively.

The point-based classification technique produced various overall accuracies for land cover classification. Overall accuracies of 77.8%, 92.7% and 88.0% were achieved when using NDVI_NIR-MIR_, NDVI_NIR-G_ and NDVI_MIR-G_, respectively. As previously mentioned, the unclassified points and the ground filtering are two sources of classification errors. The error of the unclassified points ranges from 0.0–2.3%, while the ground filtering errors range from 0.0–4.4%. 

With the focus on the individual classes, about 35.6% of the tree points were omitted (64.4% producer accuracy), and those points were wrongly classified as buildings when NDVI_NIR-MIR_ was used. This omission caused a misclassification of the buildings class with about 37% (63% user accuracy). This is primarily due to the moisture content of the vegetation, where dry vegetation exhibits high intensity values in C1 [38]. As a result, the NDVI_NIR-MIR_ yielded low values, and hence, the tree points were misclassified as buildings. Similarly, about 19.7% of the grass points were omitted (80.3% producer accuracy) and mainly classified as roads. Furthermore, about 31.1% and 14.5% of roads points were misclassified as grass when NDVI_NIR-MIR_ and NDVI_MIR-G_, respectively, were used. 

In addition, about 20.7% (79.3% producer accuracy) of the buildings’ points were wrongly classified as trees when NDVI_MIR-G_ was used. This is mainly because some roof materials exhibit high intensity values in C1, and hence, the NDVI_MIR-G_ increased, leading to the classification errors. As a result, this omission caused a misclassification of the tree class for about 14.4% (85.6% user accuracy).

Although previous attempts showed that radiometric correction and normalization can lead to the improvement of intensity homogeneity [11,12,13], such phenomena cannot be observed in this research. This might be attributed to the fact that the multispectral LiDAR data, used in this research, were collected with a narrow scan angle, whereas the LiDAR data of the aforementioned studies were collected with a wide scan angle that caused obvious energy loss especially close to the edge of the scan line. In addition, the target-related parameters, including the range to the target, the target size, the laser beam incident angle and the illumination of the target material should not necessarily have similar influence on the LiDAR intensity data to the previous studies. Another reason is that the environmental parameters related to the collected data, such as aerosol and Rayleigh scattering, aerosol absorption and atmospheric attenuation, are not changed over the study area, and hence, they do not have the same effect on the LiDAR intensity data as the previous studies. Furthermore, the three channels of the Optech Titan sensor are well controlled by its in-house transfer function, where the recorded signal strength is linear and stable. As such, there is no significant loss of energy due to the signal transmission function. Consequently, radiometric correction may not always be required under similar conditions.

## 7. Conclusions

This research discussed the use of multispectral LiDAR data in land cover classification of an urban area. The multispectral data were collected by the Optech Titan sensor operating at three wavelengths of 1550 nm, 1064 nm and 532 nm. Two classification techniques were used to classify the multispectral LiDAR data into buildings, trees, roads and grass. The first technique is image-based classification, where the LiDAR intensity and height data were converted into images. Two band combinations were stacked including combined three-intensity images and combined three-intensity images with DSM. The maximum likelihood classifier was then applied to the two band combinations. This technique achieved an overall classification accuracy of about 77.32% from the LiDAR intensity data only. The classification accuracy was improved to 89.89% when DSM was incorporated with the LiDAR intensity data. 

The second technique is point-based classification, where the 3D LiDAR points in the three channels were combined and three intensity values were assigned to each single LiDAR point as a pre-processing step. Ground filtering, using skewness balancing and slope-based change, was then applied to separate the LiDAR data into ground and non-ground points. Subsequently, the NDVIs were computed and threshold values were estimated using Jenks break optimization method to cluster the ground points into roads and grass, and the non-ground points into buildings and trees. This technique achieved overall accuracies of 77.83%, 92.70% and 88.02% when NDVI_NIR-MIR_, NDVI_NIR-G_ and NDVI_MIR-G_ were used, respectively. A physical model based on the radar range equation was used for radiometric correction of the intensity data. The correction considered the system parameters and the topographic effect. It has been noticed that there is no significant effect on the land covers’ classification results after applying the radiometric correction on this particular dataset. The presented work demonstrates the advantage of using multi-dimensional LiDAR data (intensity and height) over a single dimensional LiDAR data (intensity or height).

## Figures and Tables

**Table 1:** Optech Titan sensor specifications.

Parameter	Specification
Wavelength	Channel 1 = 1550 nm, Channel 2 = 1064 nm, Channel 3 = 532 nm
Altitude	Topographic: 300–2000 m above ground level (AGL), all channels Bathymetric: 300–600 m AGL, Channel 3
Scan Angle (FOV)	Programmable; 0–60° max
Beam Divergence	Channels 1 and 2 = 0.35 mrad, Channel 3 = 0.7 mrad
Pulse Repetition Frequency	50–300 kHz/channel; 900 kHz total
Scan Frequency	Programmable; 0–210 Hz
Swath Width	0–115% of AGL
Point Density ^1^	Bathymetric: >15 points/m^2^ Topographic: >45 points/m^2^

^1^ Assumes 400 m AGL, 60 m/s aircraft speed, 40° FOV.

**Table 2:** Reference points for the four classes.

Class	Buildings	Trees	Roads	Grass	Total
Number of Points	12,253	17,740	4566	11,059	45,618

**Table 3:** Confusion matrix for CIBs.

Classification Data	Reference Data				Total Row	User’s Accuracy (%)
	Buildings	Trees	Roads	Grass		
Buildings	7910	359	341	96	8706	90.86
Trees	4153	15,346	857	1895	22,251	68.97
Roads	157	1432	3319	370	5278	62.88
Grass	33	603	49	8698	9383	92.70
Total column	12,253	17,740	4566	11,059	45,618	
Producer’s Accuracy (%)	64.56	86.51	72.69	78.65		

Overall accuracy: 77.32%; overall Kappa statistic: 0.675.

**Table 4:** Confusion matrix for CIBs_DSM.

Classification Data	Reference Data				Total Row	User’s Accuracy (%)
	Buildings	Trees	Roads	Grass		
Buildings	11,550	637	254	110	12,551	92.02
Trees	583	16,969	336	2236	20,124	84.32
Roads	78	125	3926	154	4283	91.66
Grass	42	9	50	8559	8660	98.83
Total column	12,253	17,740	4566	11,059	45,618	
Producer’s Accuracy (%)	94.26	95.65	85.98	77.39		

Overall accuracy: 89.89%; overall Kappa statistic: 0.855.

**Table 5:** Threshold values (*NDVI_thrd*).

	Non-Ground Points	Ground Points
NDVI_NIR-MIR_	−0.026	−0.035
NDVI_NIR-G_	0.314	0.288
NDVI_MIR-G_	0.373	0.354

**Table 6:** Confusion matrix for point classification based on NDVI_NIR-MIR_.

Classification Data	Reference Data				Total Row	User’s Accuracy (%)
	Buildings	Trees	Roads	Grass		
Unclassified	1	25	104	76	206	
Buildings	11,013	6283	120	67	17,483	63.0
Trees	1212	11,432	19	155	12,818	89.2
Roads	4	0	4175	1878	6057	68.9
Grass	23	0	148	8883	9054	98.1
Total column	12,253	17,740	4566	11,059	45,618	
Producer’s Accuracy. (%)	89.9	64.4	91.4	80.3		

Overall accuracy: 77.8%; overall Kappa statistic: 0.695.

**Table 7:** Confusion matrix for point classification based on NDVI_NIR-G_.

Classification Data	Reference Data				Total Row	User’s Accuracy (%)
	Buildings	Trees	Roads	Grass		
Unclassified	8	285	74	44	411	
Buildings	11,212	734	124	14	12,084	92.8
Trees	1009	16,721	21	174	17,925	93.3
Roads	1	0	4200	670	4871	86.2
Grass	23	0	147	10,157	10,327	98.4
Total column	12,253	17,740	4566	11,059	45,618	
Producer’s Accuracy (%)	91.5	94.3	92.0	91.8		

Overall accuracy: 92.7%; overall Kappa statistic: 0.897.

**Table 8:** Confusion matrix for point classification based on NDVI_MIR-G_.

Classification Data	Reference Data				Total Row	User’s Accuracy (%)
	Buildings	Trees	Roads	Grass		
Unclassified	19	447	22	59	547	
Buildings	9722	1016	118	35	10,891	89.3
Trees	2502	16,277	82	160	19,021	85.6
Roads	1	0	4027	680	4708	85.5
Grass	9	0	317	10,125	10,451	96.9
Total column	12,253	17,740	4566	11,059	45,618	
Producer’s Accuracy (%)	79.3	91.8	88.2	91.6		

Overall accuracy: 88.0%; overall Kappa statistic: 0.831.
